# Supplementary figures and images for: Chemically Defined Conditions Mediate an Efficient Induction of Dental Pulp Pluripotent-Like Stem Cells into Hepatocyte-Like Cells
Source: Stem Cells Int. 2021 Nov 8;2021:5212852. doi: 10.1155/2021/5212852 (PMC8593589; doi:10.1155/2021/5212852)

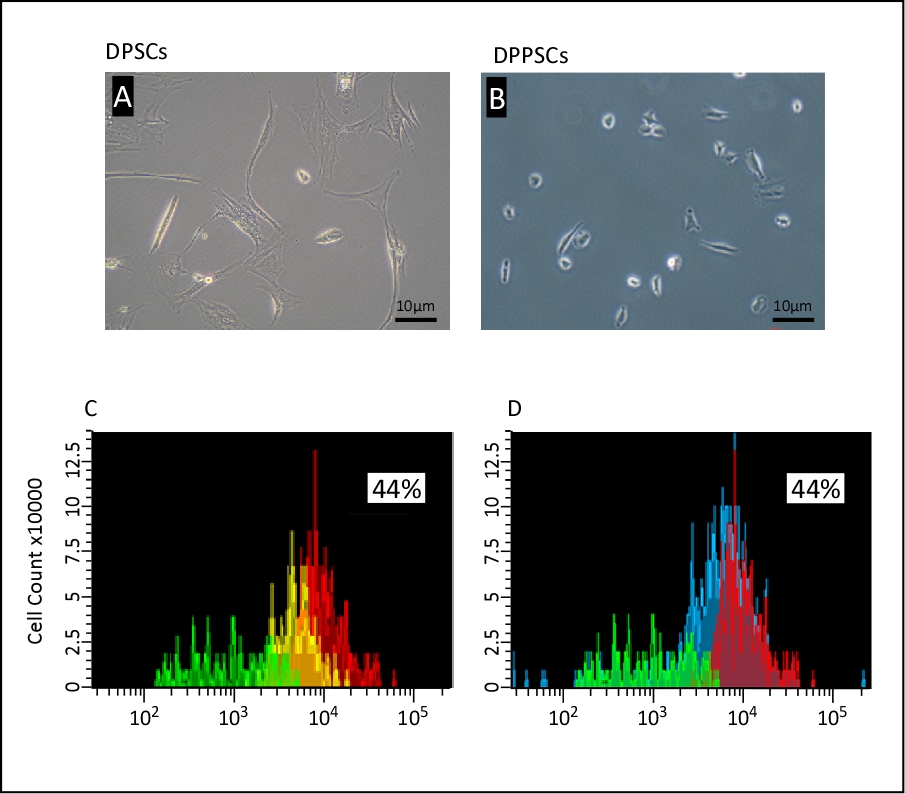

Supplement: Supplementary 1 — Supplementary Figure 1: morphological differences between DPSCs and the subpopulation DPPSCs. (A, B) Representative images for DPSC population and isolated DPPSC subpopulation, respectively, showing morphological differences between the two cell populations. (C, D) Representative image for flow cytometry assay showing the expression of GATA4 (green) and AFP (red) markers (yellow color represents cells that overexpress both markers) of DPPSC differentiated to hepatocyte-like cells at day 14. Hoechst nuclear marker in blue. [file 5212852.f1.jpg]

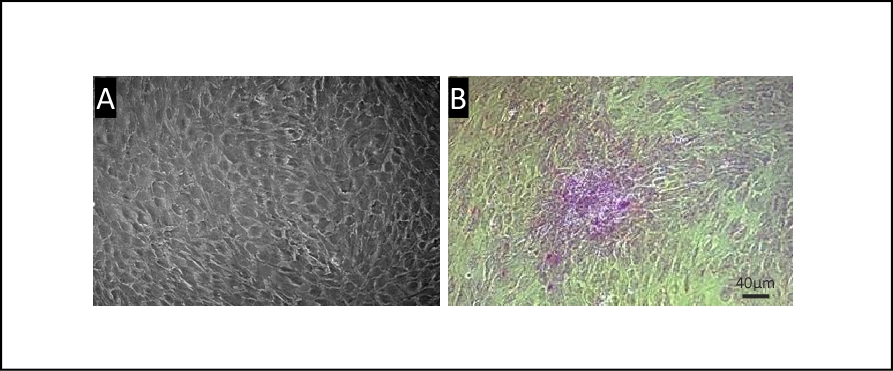

Supplement: Supplementary 2 — Supplementary Figure 2: DPPSC-mediated hepatocyte-like cells at differentiation day 22 stained. (A) PAS-unstained cells and (B) PAS-stained cells indicate the ability to store glycogen at this differentiation day. The generated hepatocyte-like cells possess a semipolygonal shape. [file 5212852.f2.jpg]
